# The relationship between basal and squamous cell skin cancer and smoking related cancers

**DOI:** 10.1186/1756-0500-4-556

**Published:** 2011-12-22

**Authors:** Freddy Sitas, Xue Qin Yu, Dianne L O'Connell, Leigh Blizzard, Petr Otahal, Leah Newman, Alison Venn

**Affiliations:** 1 Cancer Research Division, Cancer Council New South Wales, Sydney, Australia; 2 Menzies Research Institute, University of Tasmania, Hobart, Australia

## Abstract

**Background:**

We compared the risk of being diagnosed with smoking-related cancers (lung, oral cavity, upper digestive and respiratory organs, bladder, kidney, anogenital cancers and myeloid leukaemia) among people with squamous cell carcinoma (SCC) or basal cell carcinoma of the skin (BCC), with risks found in the general population using data from an Australian population-based cancer registry.

**Methods:**

People diagnosed with BCC or SCC in 1980-2003 reported to the Tasmanian Cancer Registry, Australia, were followed-up by linkage within the registry, until diagnosis of a subsequent smoking-related cancer, death, or until 31 December 2003. Risk of developing a future smoking-related cancer was assessed using age Standardised Incidence Ratios (SIR).

**Results:**

People diagnosed with SCC had an increased risk of lung cancer (men: SIR = 1.89, 95% confidence interval: 1.61-2.21; women: SIR = 2.04, 1.42-2.83) and all other smoking-related cancers (men: SIR = 1.38, 1.19-1.60; women: SIR = 1.78, 1.34-2.33). Men with BCC had a significant increased risk of lung cancer (SIR = 1.26, 1.10-1.44) but not of any of the other smoking-related cancers (SIR = 1.09, 0.97-1.23).

**Conclusions:**

Individuals with a history of SCC having an increased risk of developing smoking related cancers cancer suggests smoking as a common etiology. The relationship between BCC and smoking-related cancers is less certain.

## Background

Non-melanoma skin cancer (NMSC) is the most common cancer in Australia, with an estimated 374,000 new cases in 2002 [1], comprising 32% squamous cell carcinoma (SCC) and 68% basal cell carcinoma (BCC). Although exposure to sunlight is regarded as the most important risk factor for the majority of NMSC, the role of smoking in the etiology of these cancers, especially SCC has been unclear. There is some biological plausibility for this association, because certain tar by-products of smoking have been known since the early 20th century to cause skin cancers in experimental animals and humans [2].

It is well established that tobacco smoking is causally related to a number of squamous cell cancer subtypes in several internal organs including those of the oral cavity, oesophagus, lung, bladder and cervix [3]. With respect to the skin, it is inconclusive whether smoking is also related to SCC [4]. The relationship between smoking and risk of SCC has been found in a number of case control [5,6] and cohort studies [7,8], but no association was found between smoking and BCC [6,7]. Current smokers, compared with never smokers, were at a higher risk of SCC with odds ratios (or relative risks) ranging from 2.9 to 3.9 in different populations [5-7]. If smoking is associated with an increased risk of SCC, then it would be expected that those with SCC would be at a higher risk of lung and other tobacco associated cancers. Despite a number of studies in a systematic review supporting this association [9], others found that history of a previous diagnosis of SCC was associated with a reduced risk of subsequent cancer including some smoking-related cancers [10,11]. The possible reasons for these differences are varied and unclear. Hence the epidemiological evidence of an association between a previous diagnosis of SCC and a subsequent smoking-related cancer remains unclear.

In this study, we compared the risk of developing a future smoking-related cancer among people with SCC and BCC with that of the general population of Tasmania, Australia. The population-based Tasmanian Cancer Registry (TCR) is one of the few registries worldwide that collects information on histologically confirmed NMSC. The availability of these data and other incident primary cancers from the TCR provided us a unique opportunity to assess whether people with either SCC or BCC have an excess risk of lung and other smoking-related cancers.

## Methods

### Study population

Tasmania is an island State which lies south of the mainland of Australia, between latitudes 40 and 43 degrees south with a uniform moderate ultraviolet (UV) index across the island of around 5 http://www.bom.gov.au/jsp/ncc/climate_averages/uv-index/index.jsp, and corresponding homogeneous melanoma incidence rates [12]. It has a population of approximately 460,670 people in 2001 [13], predominantly of Caucasian origin. By statutory regulation the TCR (established in 1977) receives compulsory cancer notifications from all Tasmanian pathology laboratories and also notifications from pathology laboratories of other Australian States and Territories if people have been identified as Tasmanian residents [14]. Cancer notifications are also supplied by the two radiation oncology clinics and private and public hospitals in Tasmania [14]. The TCR generally has high standards of data completeness, quality and follow-up, with over 80% of lung cancers verified microscopically; the data are accepted by the International Agency for Research on Cancer for publication in Cancer Incidence in Five Continents [14]. The TCR is the only population-based registry in Australia that records histologically confirmed NMSC, mainly BCC and SCC [15]. All people aged 40-74 years diagnosed with BCC or SCC reported to the TCR between 1980 and 2003 were followed-up, by record-linkage, until diagnosis of a subsequent lung cancer (C33-C34) or other smoking related cancer, death, or 31 December 2003. Other smoking-related cancers are those defined by IARC in 2004: the oral cavity (C01-C06, C09), upper digestive (C15, C16, C22, C25) and respiratory organs (C10-C14, C32), bladder (C67), kidney (C64-C65), anogenital cancers (C21, C51-C53, C60), and myeloid leukaemia (C42) [3]. Age standardised (world) lung cancer incidence rates are 35 per 100,000 for males and 21 per 100,000 for females, all the other smoking related cancers are rarer [14]. The study was approved by the Human Research Ethics Committee (Tasmania) Network.

### Statistical analysis

To calculate person-years at risk for the cohort of BCC or SCC cases, the time-at-risk started at the first BCC or SCC diagnosis in or after 1980 and ended at the diagnosis of the first primary smoking-related cancer, death or the end of 2003, whichever occurred first. Deaths in the cohort were determined by linkage with records from the Registry of Births, Deaths and Marriages and the National Death Index [16]. Age, sex and period-specific cancer incidence rates in Tasmania were used to calculate the expected numbers of lung or other smoking-related cancers in those with BCC or SCC. The Standardised Incidence Ratio (SIR) was calculated as the ratio of the observed to the expected numbers of people with a previous diagnosis of BCC or SCC who had a subsequent smoking-related cancer. A 95% confidence interval (CI) for the SIR was calculated by assuming a Poisson distribution for the observed number of people with smoking-related cancers among those with BCC or SCC [17]. Absolute excess risk (AER) of a subsequent smoking-related cancer was also calculated by subtracting the expected number of cases in the general population from those observed in the cohort of BCC or SCC, and expressed in relation to the number of person-years at risk [18]. Because smoking prevalence is different for males and females, we estimated the SIRs and AERs separately by sex. Data were analysed using SAS release 9.1 (SAS Institute, Cary, NC).

## Results

A total of 9,086 SCC and 20,073 BCC cases were included in this study with mean age at diagnosis being 62.0 and 57.5 years for SCC and BCC respectively. During follow-up through to the end of 2003, 197 subsequent lung cancers and 233 other smoking-related cancers occurred in the SCC patients and 278 lung cancers and 391 other smoking-related cancers occurred among BCC patients. Median follow-up time was 4.13 years for SCC cases and 5.60 years for BCC cases.

People diagnosed with SCC had an increased risk of lung cancer (SIR = 1.89, 95% CI: 1.61-2.21 for men; SIR = 2.04, 1.42-2.83 for women) and increased risks for all other smoking-related cancers (SIR = 1.38, 1.19-1.60 for men; SIR = 1.78, 1.34-2.33 for women) (Table 1). Although there were relatively fewer cases of smoking-related cancers among women, a significant association was found.

**Table 1:** Risk of subsequent smoking-related cancers after non-melanoma skin cancer diagnosed at age 40-74 years in 1980-2003, Tasmania, Australia

	Number of observed cases	Person-years	Standardised Incidence Ratio	95% confidence intervals	AER*
**SCC**					

Lung cancer					

Men	162	31,612	1.89	1.61-2.21	2.41

Women	35	15,800	2.04	1.42-2.83	1.13

All other smoking-related cancers					

Men	179	31,673	1.38	1.19-1.60	1.56

Women	54	15,861	1.78	1.34-2.33	1.49

**BCC**					

Lung cancer					

Men	221	77,379	1.26	1.10-1.44	0.60

Women	57	57,431	1.14	0.86-1.47	0.12

All other smoking-related cancers					

Men	293	77,455	1.09	0.97-1.23	0.32

Women	98	57,550	1.10	0.89-1.34	0.15

* Absolute excess risk per 1,000 person-years

Among people with BCC, only men had a significantly increased risk of lung cancer (SIR = 1.26, 1.10-1.44) but the association with all other smoking-related cancers was not statistically elevated (SIR = 1.09, 0.97-1.23).

The absolute excess risk (AER) for smoking-related cancers among SCC cases was greater than for BCC cases and greater among men than women.

## Discussion

To our knowledge, this study is the first in Australia to compare the risk of smoking-related cancers among people with BCC or SCC with that of the general population. Compared with people in the general population, those with a history of SCC had approximately a two-fold increased risk of developing lung cancer (and an AER of 1.98 per 1,000 person-years) and about 40-80% increased risk of developing all other smoking-related cancers (and an AER of 1.54 per 1,000 person-years). In contrast, although men with BCC had a significantly increased risk of lung cancer compared with that of the general population, the excess risk was lower than that for men with SCC.

This is a population-based study with up to 24 years of follow-up for people of predominately Caucasian origin with BCC or SCC in a well-defined geographical region, with minimal variation in UV exposure and melanoma rates. In addition, the fact that all BCC and SCC and over 80% of subsequent primary cancer diagnoses were pathologically confirmed [14] reduces potential misclassification of diagnosis of BCC, SCC or subsequent smoking-related cancers.

Our results, suggesting an increased risk of lung cancer and other smoking-related cancers among people with SCC, add to the evidence supporting the association between SCC and subsequent smoking-related cancers. Frisch et al. (1995) first reported, using Danish Cancer Registry data, that people with SCC were at higher risk of a subsequent diagnosis of a number of smoking-related cancers including cancers of the lung, lip, pharynx, larynx, salivary glands and leukaemia [19]. Increased risks were also found for cancers of the nasal cavities, penis and vulva/vagina but these did not reach statistical significance due to small numbers [19]. Since then this association has been confirmed in European [20-24] and North American [25-27] populations and in both registry-based studies [20-25] and cohort studies [26,27]. A recent systematic review [9] provided strong and consistent evidence supporting an association between SCC and lung cancer and individual smoking-related cancers of the salivary glands, lip, mouth and pharynx, and myeloid leukaemia. Our study is unique in that it is the first study of the association between SCC and subsequent smoking-related cancers in the Southern hemisphere, and where the UV index level is higher (UV index in Tasmania: around 5) than in most European and North American countries (UV Index: 1 to 3) where most previous studies were conducted.

A potential source of bias is that those with a history of NMSC diagnosis may have had more regular medical check-ups, particularly after SCC [7], thus increasing the likelihood of detection of subsequent cancers compared with the general population. However it is unlikely that increased detection would be biased towards any particular cancer type. Another source of bias might be that we could not exclude prevalent NMSC at the time of inception of the cohort, however the prevalence for 'southern' Australia is expected to be less than 1% [1]. Another limitation is incomplete coverage of BCC and SCC cases because cases were notified to the TCR only if the diagnosis had been histologically confirmed or if the treatment had been provided at one of the two radiation oncology clinics in the State [28]. However without histological confirmation, diagnostic certainty of SCC or BCC would be poorer and have the potential to dilute any putative associations.

## Conclusions

We found that in Tasmania, Australia, people with SCC had a significantly elevated risk of being diagnosed with lung cancer and other smoking-related cancers. Risk factors in common for these two types of cancers, including exposure to UV, smoking, and lifestyle factors such as socioeconomic status, may explain some of the findings. UV exposure is the main risk factor for SCC and may influence the subsequent cancers such as non-Hodgkin lymphoma (NHL) and leukaemia through immunosuppression and reduced DNA repair capacity; however, NHL is not included in the smoking-related cancers and leukaemia contributes only a small number to that group. Incidence rates of SCC in Tasmania vary by socioeconomic status (A.V. Unpublished data) which might confound the association between SCC and smoking-related cancers. However, the association between smoking and SCC remained after controlling for several potential confounding factors in a number of cohort studies [26,27] which indicates that these factors may not change the main finding of this study. Therefore, smoking seems the most likely shared aetiological factor for these conditions. Literature from case control [5,6] and cohort [7] studies suggest that current smokers had about three to four-fold increased risk, compared with never smokers, for developing SCC. Furthermore, in three studies where a dose response was measured, the risk of SCC increased in relation to the number of cigarettes currently smoked and is lower for ex-smokers [5-7]. The association between smoking and SCC is therefore well worth investigating in Australia. In the meanwhile, encouraging individuals with SCC who smoke to quit smoking may reduce the risk of subsequent smoking-related primary cancer.

## Abbreviations

BCC: Basal Cell skin cancer; AER: Absolute excess risk; CI: confidence interval; NHL non-Hodgkin lymphoma; NMSC: Non-melanoma skin cancer; SCC: Squamous cell skin cancer; SIR Standardised incidence ratio; TCR: Tasmanian Cancer Registry; UV: Ultraviolet.

## Competing interests

The authors declare that they have no competing interests.

## Authors' contributions

FS and AV conceived the study, DO'C and XQY assisted with further refinement of the study protocol, XQY did the data analysis with assistance from DO'C, LB and PO, XQY prepared the first draft of the manuscript; FS, DO'C, LB, PO, LN and AV revised the manuscript critically. All authors read and approved the final version of the manuscript.
